# Strawberries and Cream: The Relationship Between Food Rejection and Thematic Knowledge of Food in Young Children

**DOI:** 10.3389/fpsyg.2021.626701

**Published:** 2021-02-16

**Authors:** Abigail Pickard, Jean-Pierre Thibaut, Jérémie Lafraire

**Affiliations:** ^1^Center for Food and Hospitality Research, Cognitive Science, Institut Paul Bocuse Research Center, Lyon, France; ^2^LEAD-CNRS UMR5022, University of Burgundy, Dijon, France

**Keywords:** categorization, taxonomic categories, thematic relations, food cognition, food rejection, food neophobia, conceptual representation

## Abstract

Establishing healthy dietary habits in childhood is crucial in preventing long-term repercussions, as a lack of dietary variety in childhood leads to enduring impacts on both physical and cognitive health. Poor conceptual knowledge about food has recently been shown to be a driving factor of food rejection. The majority of studies that have investigated the development of food knowledge along with food rejection have mainly focused on one subtype of conceptual knowledge about food, namely taxonomic categories (e.g., vegetables or meat). However, taxonomic categorization is not the only way to understand the food domain. We also heavily rely on other conceptual structures, namely thematic associations, in which objects are grouped because they share spatial-temporal properties or exhibit a complementary relationship (e.g., soft-boiled egg and soldiers). We rely on such thematic associations between food items, which may not fall into the same taxon, to determine the acceptability of food combinations. However, the development of children's ability to master these relations has not been systematically investigated, nor alongside the phenomenon of food rejection. The present research aims to fill this gap by investigating (i) the development of conceptual food knowledge (both taxonomic and thematic) and (ii) the putative relationship between children's food rejection (as measured by the Child Food Rejection Scale) and both thematic and taxonomic food knowledge. A proportional (A:B::C:?) analogy task, with a choice between taxonomic (i.e., bread and pasta) and thematic (i.e., bread and butter) food associates, was conducted on children between 3 and 7-years-old (*n* = 85). The children were systematically presented with either a thematic or taxonomic food base pair (A:B) and then asked to extend the example type of relation to select the respective thematic or taxonomic match to the target (C:?). Our results revealed, for the first time, that increased levels of food rejection were significantly predictive of poorer food identification and decreased thematic understanding. These findings entitle us to hypothesize that knowledge-based food education programs to foster dietary variety in young children, should not only aim to improve taxonomic understanding of food, but also thematic relations.

## Introduction

A lack of dietary variety in childhood leads to enduring impacts on both physical and cognitive health (Evans et al., 2018). Consequently, establishing healthy dietary habits in childhood is crucial in preventing long-term repercussions (Jirout et al., 2019). Food rejection, namely food neophobia and pickiness, has been determined as a central psychological driver in reduced dietary variety in young children (Carruth et al., 2004; Levene and Williams, 2017). Food neophobia is defined as a fear of novel food stimuli or food-based situations and is often witnessed as a reluctance or unwillingness to try unfamiliar foods (Dovey et al., 2008; Lafraire et al., 2016; Crane et al., 2020). Food pickiness, on the other hand, is the rejection of both familiar or unfamiliar foods and textures (Dovey et al., 2008; Lafraire et al., 2016). Importantly, both food pickiness and food neophobia similarly account for inadequate food consumption and nutrient deficiencies in young children (Dovey et al., 2008; Lafraire et al., 2016; Rioux et al., 2017b). Longitudinal research demonstrates that, although food neophobia and food pickiness have an increased prevalence during childhood, such dietary habits and behaviors prevail well into adulthood (Nicklaus et al., 2005). Consequently, it is fundamental to identify the key driving mechanisms of food rejection in young children to tackle poor eating habits and behaviors (World Health Organization, 2014).

Food rejection, and in particular food neophobia, ultimately depends upon children's recognition and knowledge when they are confronted with a possible food source (Birch, 1979). A key cognitive mechanism enabling food recognition and related feelings of familiarity is categorization. Taxonomic categorization depicts classifying items into a hierarchical structure based on shared features or properties (e.g., edibility, overall shape, sweetness, et cetera); for example, an apple belongs to the category of fruits, which may be further categorized into the broader category of food (Lucariello et al., 1992; Murphy, 2002; Gelman, 2003). It allows us to group foods, generalizing their key properties (e.g., edibility, toxicity, et cetera) to novel objects based on category membership (Ross and Murphy, 1999; Nguyen, 2008; Lafraire et al., 2016; Rioux, 2020). For example, we rarely encounter the same apple twice, but having the category knowledge of an apple allows us to make the relevant inferences that it is similar to apples we have previously encountered (Murphy, 2002). However, foods, like many other categories, are multidimensional concepts and rely on different methods of association to form inferences (Ross and Murphy, 1999).

One such alternative method of inferring information is through thematic knowledge. Thematic relations group items based on a complementary or spatial-temporal relationship, such as a banana and a monkey because they form a complementary and well-known association (Gelman and Markman, 1986; Markman, 1989). Thematic categories display diverse types of associations, such as functional (e.g., soup and spoon), co-occurring (e.g., bread and butter), or even causal relations (e.g., cow and milk) (Keil, 1989; Markman, 1989). As such, thematic categories are useful in that they provide us with situational cues and inferences on the origin, use, and possible consequences of items, which is essential in the food domain. For example, knowing the thematic association of certain foods with a bowl, allows us to infer that when we encounter an unfamiliar substance served in a bowl it is likely to be edible. In contrast, thematic categorization has much less generalization power. Knowing that soup and spoon belong to the same thematic category does not mean that soup properties can be extended to spoon.

Nevertheless, both thematic and taxonomic knowledge in the food domain provides us with cues that enhance our recognition of food-based situations (such as meal times) that underpin food acceptance and rejection. Whilst a caregiver may present a variety of foods to a child, it is ultimately the child's decision whether to accept or reject the food. Poor category knowledge in the food domain lends itself to increased uncertainty and feelings of novelty in the food domain and children with less food knowledge may display higher levels of food rejection to avoid distaste or even potential toxicity (Nguyen and Murphy, 2003). The overarching purpose of the present study is to examine the relationship between children's food rejection (as measured by the Child Food Rejection Scale) with taxonomic and thematic food category knowledge.

To address the possible link between thematic and taxonomic food category knowledge and food rejection in young children, we must first establish at what age children acquire such an understanding of food categories. Studies of children's food knowledge indicate that 3-year-olds already display taxonomic understanding and can distinguish between food and non-food items (Bovet et al., 2005; Lafraire et al., 2016). Impressively, 3-year-old children are further capable of accurately identifying and distinguishing vegetables from fruits (Brown, 2010; Rioux et al., 2016). Another study witnessed that 3-year-olds displayed an above-average accuracy for taxonomic matching and showed a rapid development of taxonomic food knowledge between 3 and 7 years old (Nguyen and Murphy, 2003). Nguyen and Murphy (2003), using an induction task, demonstrated that 7-year-olds, and to a lesser extent even 4-year-olds, could selectively use taxonomic food categories (such as vegetables and fruits) to extend biochemical properties (i.e., similar food composition).

In contrast, studies of children's thematic category knowledge in the food domain are scant. A noticeable exception is a study conducted by Thibaut et al. (2016), which showed that both 4 and 9-year-olds were not likely to rely on thematic category knowledge to make inductive inferences about biological or psychological properties of food (i.e., do both strawberries and cream make Diddl feel ill). They instead observed that 9-year-old children referred to taxonomic category knowledge to make both biological and psychological inferences about the effects of certain foods (i.e., both broccoli and carrots make Diddl happy). In a simplified forced-choice triad version of the task, they showed that both 5 and 6-year-olds were capable of extending psychological and biological properties to taxonomic over thematic food categories. Whilst their results demonstrate that children as young as five have a taxonomic understanding of foods and can inductively infer common properties, there are no conclusive findings regarding the age at which children use thematic category knowledge to guide inference in the food domain. Therefore, studying the acquisition of thematic categories below 5 years of age is one purpose of the present experiment.

Developmental literature outside of the food domain indicates that children as young as 3 years old have a good ability to make thematic and taxonomic associations (Markman and Hutchinson, 1984; Huttenlocher and Smiley, 1987). However, they also evidence that there is a significant improvement in both abilities between 3 and 6 years old (Smiley and Brown, 1979; Waxman and Namy, 1997; Nguyen and Murphy, 2003; Nguyen, 2007). Based on these studies, we hypothesized that there is a significant development of thematic and taxonomic knowledge in children between 3 and 6 years old.

Considering that the acuteness of food rejection is concomitant with the rapid development in children's understanding and categorization, we argue that food rejection is closely intertwined with children's development of category-based understanding in the food domain (Lafraire et al., 2016; Rioux et al., 2016). Of the few studies into children's category knowledge in the food domain, it is only recently that work has investigated the possible link between food rejection and food categorization. Rioux et al.'s pivotal work demonstrated that 3 to 6-year-old children with strong food rejection tendencies displayed poorer performance in a fruit and vegetable forced-choice sorting task (Rioux et al., 2016). More specifically, the researchers witnessed that higher levels of food rejection predicted a higher rate of false alarms for fruit and vegetable categorizations. This indicates that children with high food rejection tendencies inaccurately over-categorize taxonomic groups, which possibly drives them to reject a much larger number of inaccurately categorized fruits and vegetables. In a later study, the same researchers also revealed that food rejection and taxonomic category-based induction performance were significantly negatively correlated (Rioux et al., 2017b, 2018). Whilst children with low food rejection tendencies referred to taxonomic categories when generalizing properties to unknown foods, high food-neophobic and picky-eaters relied on perceptual similarity when generalizing properties (Rioux et al., 2017b). According to the authors, food rejection tendencies restrict and reduce the learning opportunities concerning taxonomic food groups, resulting in a poorer system of taxonomic understanding, and ultimately uncertainty when confronted with unfamiliar food.

Their interpretation presents a central argument for the necessity to investigate the link between food rejection and children's knowledge of thematic categories in the food domain. Thematic categories rely heavily on previous exposure and a degree of familiarity, perhaps more so than taxonomic categories (Markman, 1989; Murphy, 2002). High food rejection tendencies may impede children's understanding of thematic relations because they restrict their interactions and experiences with the food domain. As such, all dimensions related to food knowledge, including familiarity, category knowledge, sensory experiences, etc. would be under-developed. However, to the best of our knowledge, the relationship between thematic category knowledge and food rejection has yet to be investigated. We argue that children with high levels of food rejection would have reduced learning opportunities and experience with food, subsequently impeding their knowledge of thematic relations. Therefore, we hypothesize that children with higher levels of food rejection will have a poorer understanding of thematic associations in the food domain. This lack of thematic knowledge reduces the child's feelings of recognition and understanding when confronted with a potential food source, ultimately perpetuating the cycle of food rejection.

To examine our two leading hypotheses, we developed a proportional analogy task of the type A is to B, what C is to D (D having to be discovered), to compare the development of young children's capabilities to make taxonomic and thematic associations within the food domain. Analogical reasoning is the ability to understand or produce common relational structures between two domains despite dissimilarities between the entities (Gentner, 1983; Hofstadter, 2001; Holyoak, 2012). The children are first exposed to one of the two relations of interest (either thematic or taxonomic) in the first pair of items (A and B; for example, apple and melon both belong to the taxonomic category of fruits) and then asked to extend the example type to choose either the thematic or taxonomic match to the food target image. In the above, the child might be shown an orange and would have to choose between a pineapple–taxonomic choice and a knife-thematic choice).

Analogical understanding is critical in cognition and a core process of discovery, problem-solving, categorization, and reasoning, elements that are all crucial to food decision-making (Gentner and Markman, 1997; Markman and Wisniewski, 1997). One defining feature of analogies is that they capture relational structures between items rather than the features these items share (Gentner, 1983). For example, the pairs car/road and train/railway share the same relation, i.e., “moves on,” rather than common surface features. In our context, analogy tasks lend themselves well to investigating thematic and taxonomic knowledge because relational understanding (either thematic or taxonomic) is our central issue (Kotovsky and Gentner, 1996). The children's understanding of the analogy pair, for both taxonomic and thematic conditions, will evidence if food rejection and age predict children's thematic and taxonomic knowledge in the food domain.

## Materials and Methods

### Participants

The children were recruited from an elementary school (children aged 3 to 7-years-old) in the Southeast of France. The school inspector examined the study proposal and permitted the collection of data. This study was performed in accordance with the guidelines as described in the Declaration of Helsinki and complied with international regulations for research on human subjects.

The parents/caregivers provided the consent for their child to take part through completing the consent form and the CFRS questionnaire. Oral assent, in the presence of the child's teacher, was required from all children before going with the researcher. The child was informed that they were free to stop the task and return to class at any point. 146 children between 37.6 and 81.3 months old (*M* = 55.57, *SD* = 10.02) with parental consent participated in the study. Only 85 of the 146 children, (aged between 37.6 and 81.3 months, *M* = 58.54, *SD* = 10.71) completed all elements of the study and were retained in the analysis (see section Results).

### Materials and Procedure

To collect the measures of food neophobia and food pickiness, the Child Food Rejection Scale (CFRS; Rioux et al., 2017a) was distributed to the parents/caregivers of the children prior to the main study. The CFRS is a hetero-assessment scale that was developed and validated to measure food rejection in children aged 2 to 7-years-old. Six items form a subscale measuring food neophobia and five items measure picky/fussy eating behaviors. The caregivers are asked to rate their child's eating behaviors on a 5-point Likert scale. The maximum scores, indicating high food rejection behavior, are 25, 30, and 55 for pickiness, neophobia, and global CFRS score, respectively.

#### Material Selection

To establish an idea of the food children are already exposed to, children's books, online local school menus and food studies conducted with French children (e.g., Thibaut et al., 2016) were consulted. To assess the children's basic knowledge and familiarity with the stimulus materials, we ran a pre-test with six children between 3 and 6 years old. The children were asked to label the food stimuli to determine whether the photo was recognizable (i.e., an item-recognition control measure) and to establish the most common label used by children. The labels provided from this pre-test were used to establish whether the test participants provided an accepted label in the food identification task. The children were then asked to identify the thematic/taxonomic relations existing between the standard stimuli and the thematic/taxonomic choices in each stimulus set. Photos that were not recognized or relationships that were not identified by at least the four eldest children were not included in the further pre-tests.

As commonplace with developmental studies, a pre-test with 79 adults was conducted to select the final set of stimuli. To assess the strength of the thematic relation, the adult participants were asked to score the strength of the association that exists between the target stimulus and the associated thematic match (i.e., “on a scale of 1–7, what is the strength of the association between cereal and milk?”). Following the protocol of Ross and Murphy (1999), we calculated the mean for each pair and decided to retain those with a score above 4.0 out of 7. To determine the taxonomic knowledge of foods, participants were also asked to indicate for each of the food items whether they were good examples of their respective taxonomic categories (e.g., on a scale of 1–7, how typical is a carrot of being a vegetable?). For taxonomic food groups, items ranked above 3.5 out of 7 as typical exemplars of their respective categories were selected (a lower threshold was chosen for taxonomic belonging due to lower overall mean ratings).

The finalized set of stimuli comprised: two thematic food base pairs (pancakes:chocolate sauce and ice cream:wafer cone), two taxonomic food base pairs [banana:apple (fruits) and sardines:salmon (fish)]. One thematically associated artifact example base pair (notebook:pencil), and one taxonomically associated artifact example base pair [dog:monkey (animals)] were selected for the training task. The training triad was comprised of a soccer shoe as the target, with rain boots as the taxonomic match (footwear) and a soccer ball as the thematic choice. For the test, 16 food triads (comprised of the target food, a thematic match, and a taxonomic match) were finalized (see Appendix A for a complete overview of the final test stimuli). We ran a pilot on five adults to be sure that each analogy had only one unambiguous solution. The adults made no mistakes and, thus, there was no variance in the data set.

#### Pilot Study

A pilot test was conducted on children aged between 3 and 6 years-old (*n* = 7) to see if the younger children were: (1) able to follow and understand the analogy task, (2) identify the food stimuli in the test phase, and (3) understand the thematic/taxonomic relation presented in the analogy pair. The pilot followed the procedure of the main test; the two artifact examples and the artifact triad were provided to explain the procedure, followed by the 16 test-phase trials. After each object selection, the child was asked to name all three objects in the 16 separate trials to determine whether the child was familiar with all the presented items. All of the images were correctly identified or adequately described by at least 80 percent (six of the seven children) and thus retained for the main test as per Lucariello et al. (1992).

#### Food Analogy Task

The task followed a classical analogy paradigm (e.g., Rattermann and Gentner, 1998; Goswami, 2001; Thibaut et al., 2010), where stimuli A:B::C:?, are presented and participants must select a D item from two options (thematically or taxonomically related), in such a way that the C:D pair share the same type of association as the A:B pair (either thematic or taxonomic). If the child understands the relationship between images A and B, they should then apply this relation to image C to identify the appropriate choice of D, from two possible options. Selecting the appropriate response for D implies that the child has understood the type of relationship of the analogy pair and identified the pair demonstrating the same relation. The taxonomic and thematic performance scores were thus calculated as the percentage of appropriate selections when the taxonomic or thematic example, respectively, was presented.

To standardize the photographs presented to the children, each item had to be the most typical representation and contain enough detail consistent with the real-life object, whilst not being overly complicated nor ambiguous (Snodgrass and Vanderwart, 1980). The objects in each triad of photographs were scaled to correspond with the relative dimensions the three objects would have with one another in real life (i.e., the pastry case would be larger than the strawberry). Because we pit thematic against taxonomic associations, we wanted to avoid any factors that could cause a bias toward one type of association over the other. No labels were given to any of the presented stimuli because studies show that providing the labels of items increases taxonomic responses in children (Markman and Wachtel, 1988).

Research indicates that analogical reasoning ability can vary greatly in children between 3 and 6 years old (Christie and Gentner, 2014). However, cognitive development research has successfully used analogy tasks to investigate conceptual development, relational reasoning, and problem solving with pre-verbal children (Ferry et al., 2015, with 6-month-olds and Chen et al., 1997, with 13-month-olds). However, to draw conclusions on thematic and taxonomic knowledge, it was imperative that only children capable of understanding analogies of the sort we used here were included in the analyses. Consequently, we included the training task to determine which of the participants succeeded in understanding the thematic and taxonomic analogies. The two training trials used the same triad (sneaker and soccer ball or rubber boots) to demonstrate that in each triad there are two possible relationships pitted against one another (a thematic or taxonomic match). Children who seemingly failed to identify the corresponding pair in the training were removed on the assumption that if they failed to identify the correct relationship in the analogy examples, their responses to the food trials would be at random. Of the 146 children who provided assent to participate, only 85 children successfully performed the training analogies and went on to complete all trials.

**Figure 1:**
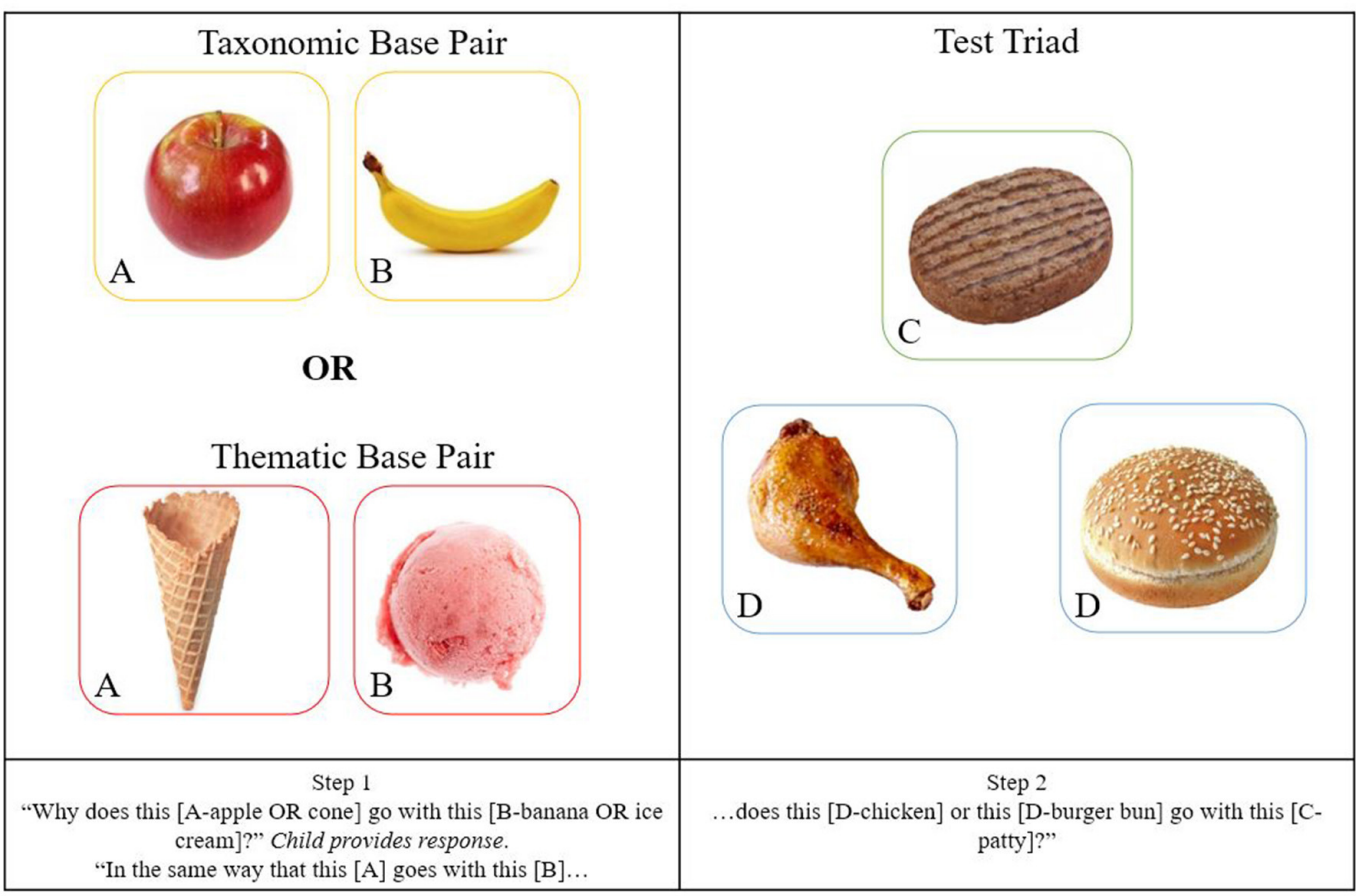
Example of stimuli presented for both taxonomic and thematic analogy condition and example test triad.

Eight trials were conducted with a thematic analogy base pair (i.e., *A* = ice cream, *B* = wafer cone) and eight trials with a taxonomic base pair (i.e., *A* = apple, *B* = banana), in a pseudo-randomized order. Then, one of the 16 triads was presented, with C (referred to as the target) (e.g., beef patty) and the respective taxonomic match (e.g., chicken) and thematic match (e.g., burger bun) for the child to select from (see Figure 1). The task instructions followed those used in previous studies with younger French children (Thibaut et al., 2016). The procedure for the taxonomic analogy condition was as such: “in the same way that this (banana) goes with this (apple), would this (chicken) or this (burger bun) go with this (patty)?” The thematic analogy condition was identical but the example pair was changed to one of the two thematic relationships, i.e., “in the same way that this (ice cream) and this (cone) go together, would this (chicken) or this (burger bun) go with this (patty)?” All 16 triads pitted a taxonomic associate against a thematic associate, but the analogy pair priming the appropriate answer alternated between taxonomic and thematic pairs in isolation.

#### Post-identification Task

The literature on categorization development frequently argues that object recognition/familiarity is important to children's categorization abilities, particularly for thematic associations (Markman, 1989; Thibaut et al., 2010). Therefore, we deemed it necessary to determine what effect children's object identification had on correct thematic and taxonomic knowledge. To measure children's familiarity with the food items, after the child had completed each trial, they were asked to label the stimuli.

#### Statistical Analyses

To determine whether age and food rejection were predictive of taxonomic or thematic performance, general linear models were computed with taxonomic and thematic analogy scores as outcomes. Due to the expected collinearity between the neophobia and pickiness variables, separate models were run with CFRS, pickiness, and neophobia, and the Akaike Information Criterion (AIC) was used as the estimator of the relative quality of the statistical models. All descriptive and inferential analyses were performed with the software R.3.5.3, and the significance level was set to 5% (*p* < 0.05).

## Results

Of the 146 children, only 85 French children completed the training analogy task and went on to complete all the test trials. 83 of the 85 participants completed the identification task of the study, two participants did not respond or were incomprehensible in their replies. The subsequent analyses are based on the 83 children (35 boys and 48 girls) who completed all training trials, test trials, and the post-identification task. The 83 children were aged between 37.6 and 81.3 months old (*M* = 58.5, *SD* = 10.7).

To calculate the performance in the food analogy task, a score of 0 was assigned if participants selected the divergent choice for each triad (e.g., thematic: taxonomic pair). In contrast, a score of 1 was assigned to each trial that the participant selected the analogous choice (e.g., thematic example: thematic choice OR taxonomic example: taxonomic choice). Scores were totaled, with a maximum possible score of eight for both thematic and taxonomic performance, and a global score of 16 for all trials collapsed together. Across all participants the scores for taxonomic and thematic performance showed similar distributions (*M* = 0.54, *SD* = 0.21; *M* = 0.57, *SD* = 0.21, respectively). As there were only two options, the children had a 50% chance of guessing the correct response, which subsequently would heavily bias the total score. Therefore, we took taxonomic performance and thematic performance as dependent variables. To account for the potential preference of either taxonomic or thematic associations, the total number of thematic and taxonomic responses for all 16 trials was calculated, regardless of the analogy pair. Four participants responded significantly above chance (12 or more responses) for the taxonomic choice and eight participants for thematic responding. Overall, there was no significant difference between the response rate for thematic or taxonomic selection regardless of the analogy example (*Z* = –0.731, *p* = ns).

To score food identification in the post-task, the responses were noted and then coded post-study (1 = correct response, 0 = incorrect response or uncertainty) based on a list of correct or synonymous labels, with a total possible score of 48. Sensory properties (e.g., sour), non-descript labels (e.g., store name), or labels that did not have 100 percent consensus among the research team, were classified as incorrect. The mean score for food identification was 75.6 percent (*SD* = 14.5%), demonstrating a good overall knowledge of the stimuli set. There were no main or interactive effects of gender or order, so these variables were not included in the subsequent analyses. There were no significant interaction effects between the variables (outside of the expected collinearity of the CFRS subscales). Across the sample, the average pickiness score was 17.7 (*SD* = 4.5) out of a possible 25, the average neophobia score was 16.69 (*SD* = 5.66).

Shapiro-Wilk analyses showed that age, pickiness, food identification, taxonomic, and thematic scores were not normally distributed, thus a Spearman's Rho matrix with bootstrapping (*B* = 1,000) was conducted to identify significant correlations between the variables (see Table 1). Age was significantly correlated with identification score (*r*_s_ = 0.510, *p* < 0.001, *N* = 83). Regression models, using a forward stepwise method were run to predict food identification from age, neophobia, and pickiness scores. Age was the sole significant predictor of food identification scores, β = 0.292, *t*(81) = 4.99, *p* < 0.001. Age explained a significant proportion of variance in food identification, *R*^2^ = 0.201, *F*(1, 81) = 20.36, *p* < 0.001.

**Table 1:** Spearman's Rho correlation matrix for all variables.

**Variable**	**1**	**2**	**3**	**4**	**5**	**6**	**7**	**8**	**9**
1. Taxonomic Score	–								
2. Thematic Score	0.053	–							
3. Total Score	0.683**	0.726**	–						
4. Identification score	−0.039	0.393**	0.262**	–					
5. Pickiness	−0.054	−0.320**	−0.240*	−0.082	–				
6. Neophobia	−0.002	−0.208	−0.162	−0.051	0.605**	–			
7. CFRS	−0.040	−0.247*	−0.207	−0.051	0.843**	0.922**	–		
8. Age	0.078	0.138	0.166	0.510**	−0.088	0.103	0.020	–	
9. Taxonomic responses	0.645**	−0.685**	−0.044	−0.311**	0.203	0.158	0.157	−0.035	–
10. Thematic responses	−0.655**	0.682**	0.038	0.298**	−0.197	−0.176	−0.168	0.051	−0.994**

** P < 0.01,

* *P < 0.05*.

Taxonomic performance did not show any significant correlations with the predictor variables and was subsequently not included in further modeling.

The most important result was a significant negative correlation between pickiness and thematic performance (*r*_s_ = −0.320, *p* = 0.003, *N* = 83) and CFRS and thematic performance (*r*_s_ = −0.247, *p* = 0.024, *N* = 83). Whereas, correct food identification was positively correlated with thematic performance (*r*_s_ = 0.393, *p* < 0.001, *N* = 83). The highest quality model, as deemed by the AIC values, indicated that global food rejection (pickiness and food neophobia totalled) showed greater statistical relevancy than food neophobia or pickiness individually. Both food identification score and food rejection score (CFRS) explained a significant proportion of variance in thematic performance *R*^2^ = 0.237, *F*(2, 80) = 12.39, *p* < 0.001 (see Table 2 for model coefficients). Food identification accounts for 15.1 percent of the variability in thematic performance; as food identification increased thematic performance increased. Whereas, CFRS accounts for 8.5 percent of the variance in thematic performance; the greater the food rejection tendencies the worse the performance for thematic associations.

**Table 2:** Regression model predicting thematic performance.

	**B**	**Std. error**	**β**	**AIC**
Step 1				−245.288
	Constant	1.144	0.924	
	Identification	0.095	0.025	0.389**
Step 2				−249.492
	Constant	3.16	1.11	
	Identification	0.091	0.024	0.373*
	CFRS	−0.055	0.018	−0.293*

*R2 = 0.151 for Step 1, ΔR2 = 0.085 for Step 2 (p = 0.014)*.

** p < 0.001,

* *p < 0.05*.

## Discussion

Leading on from the seminal work of Rioux et al. (2016, 2017b, 2018), this study aimed to investigate the development of taxonomic and thematic food knowledge in children aged 3 to 6 years. We also investigated the unexplored relationship between children's thematic knowledge and food rejection tendencies (food neophobia/picky-eating). We hypothesized that there is a significant development of food identification and both thematic and taxonomic food knowledge in children between 3 and 6 years old. We further hypothesized that food rejection tendencies (food neophobia and food pickiness) would predict poorer knowledge of thematic and taxonomic relations in the food domain.

### Development of Thematic and Taxonomic Food Knowledge

Based on previous work by Thibaut et al. (2016) and Rioux et al. (2017b), we hypothesized that even 3-year-old children would be able to thematically and taxonomically associate food. However, we also expected to witness that there would be a developmental improvement in both abilities. Our results confirm that young children are indeed capable of identifying thematic and taxonomic food relations. We witnessed that children as young as 38.8 and 40.1 months were capable of performing significantly above chance in both the thematic and taxonomic conditions, respectively. Our results are consistent with previous studies evidencing that 3-year-old children succeed above chance in determining taxonomic and thematic food associations (Nguyen and Murphy, 2003; Rioux et al., 2016; Thibaut et al., 2016).

Surprisingly, age was not directly correlated with thematic and taxonomic performance, but it was positively correlated with correct food identification. Furthermore, improved food identification was a significant predictor of better knowledge of thematic relations. These findings indicate that the relationship between age and thematic categorization ability is potentially mediated by food identification. If the child is unable to identify the foods, subsequent understanding of the thematic relation is inhibited. This appears intuitive to our understanding of thematic associations, in that these pairs depend heavily on previous exposure and require familiarity with both items and/or context of occurrence more so than taxonomic pairs (Gelman and Markman, 1986; Markman, 1989; Gelman, 2003). Further studies with a larger sample size would allow mediatory analyses to delineate the specific relationship that object identification has on age and thematic categorization performance.

### Food Rejection Is a Significant Predictor of Thematic Categorization Performance

The ultimate objective of this study was to establish how the previously reported negative relationship between food rejection and taxonomic categorization ability (Rioux et al., 2016, 2018) extends to thematic categorization ability in young children. We hypothesized that children with higher levels of food rejection would display poorer knowledge of taxonomic and thematic associations in the food domain. Our results are the first piece of evidence in the field to demonstrate that children with higher scores of food rejection show significantly worse thematic categorization performance.

We believe that poor thematic categorization is subsequent to a lack of exposure to different foods and associations, perhaps to a greater extent than the understanding of taxonomic relations. Thematic relations require the correct identification of the relationship between two items, whereas taxonomic understanding requires identifying the correct taxonomic belonging of an individual item. To learn taxonomic relations it is sufficient to view two objects (both belonging to the same taxon) individually and still have the capability to understand the shared relation. However, thematic relations, namely common food pairings, require concomitant exposure in that both stimuli must be presented at the same time to infer that they share a relation (Markman, 1989). For example, having viewed two different vegetables on separate occasions we may still conclude that they share a taxonomic relationship. However, we would only be able to understand the thematic relation of bread and butter after witnessing the two simultaneously served together. This line of reasoning would indicate that because foods involved in thematic relations are not always paired with their counterparts (i.e., bread may be served without butter), the exposure for thematic food relations is reduced to that of taxonomic relations.

Furthermore, a common trait of picky-eating behavior is the dislike of foods being paired or mixed (Carruth et al., 1998), hence, for a picky-eater several common thematic relations would not be considered thematic since they are less likely to have foods served or consumed together. Similarly, parents of neophobic children may be less likely to serve a variety of food to their child and may stick with “safe” foods and “safe” thematic combinations, reducing the child's possibility to develop knowledge of common food associations. This lack of exposure for children with food rejection tendencies may perpetuate the uncertainty of food associations, ultimately reinforcing their fear of novel food situations. Thus, the cycle of unfamiliarity and food rejection endures and learning opportunities remain decreased. Our interpretation would suggest that continuing to expose neophobic and picky-eaters to a variety of food associations would boost their understanding of thematic relations in the food domain and consequently foster food acceptance.

### Limitations and Future Research

The findings of Rioux et al.'s previous work investigating taxonomic knowledge and food rejection in young children were not replicated in our research. As we used a forced-choice paradigm pitting thematic against taxonomic matches, children had a 50:50 chance of responding correctly, regardless of having understood the categorization. Therefore, if children with high food rejection fail to identify the thematic relation, as indicated by our results, they may default to an alternative process of selection, such as perceptual similarity. Alternatively, it might be that they chose the incorrect one because it was more attractive or preferred. Choosing an incorrect option does not necessarily mean that they have not understood the targeted relation. However, future studies should attempt to delineate the default strategies children use when unable to identify the correct association.

One may also argue that taxonomic associations were apparent in both conditions of the task, as the child could have easily reasoned that two items were paired because they both belonged to the superordinate category of “foods.” Previous studies have even indicated that children may display an intermediary level of categorization between taxonomic and thematic categories, for objects that children group as the same sort, but also because of the context in a given schema or script (e.g., foods eaten at a party; Lucariello and Nelson, 1985). Literature also argues that superordinate taxonomic categories may even be considered thematic in nature based on functional/interactional relations (i.e., food gives humans energy) (Lakoff, 2008). As well as highlighting the need to decipher the nature of the relationship between thematic understanding and food rejection, our research speaks in favor of further conceptualization of thematic associations.

After reviewing previous studies on thematic associations, it appears that thematic associations are heterogeneous in nature. In the present study, we concentrated on thematically related food pairs such as “ice cream and wafer cone.” However, there are diverse thematic relations involving foods, such as foods and utensils (e.g., watermelon and a knife), or foods in certain scripts (e.g., cereal for breakfast). Some thematic pairs may be of a spatial and temporal nature (i.e., sausages and mashed potato being eaten for the same meal), whilst others may be functional (ice cream goes in a wafer cone to facilitate eating). Furthermore, thematic associates appear to be culturally bound more so than taxonomic associates are (Markman, 1989). Whilst we were not able to capture the demographics of the participants, future studies should certainly include measures of cultural and social variables. Our results also underscore an important feature of thematic and taxonomic associations, in that the former may necessitate object familiarity/identification whereas the latter may not. The relationships we witnessed between age, food identification, and thematic performance were not apparent in the taxonomic performance. Previous taxonomic studies using novel stimuli have similarly demonstrated that object identification is not a necessity of taxonomic sorting, even with children as young as 3 years old (Liu et al., 2001). In contrast, researchers have previously outlined that thematic associations are heavily dependent upon previous experience (Markman, 1989). A child that correctly labels an object is more likely to have encountered that object and as such, more likely to have witnessed the object's thematic association. It appears that having a conceptual knowledge of an object aids the understanding of thematic associations. This finding paves the way for a clearer conceptualization of thematic associations and how children come to acquire thematic understanding in the food domain.

### Conclusion

This study offers exploratory insight into the previously untold relationship between thematic food categorization ability and food rejection in children aged 3 to 6-years-old. Our study provides novel evidence that food rejection significantly predicts thematic knowledge in a food-based analogy task. In addition, age was found to be positively related to food identification ability, which was a significant predictor of improved thematic categorization performance. We propose that food rejection may cause a decreased exposure to thematic food associations, which subsequently restricts children's conceptual development of thematic relations. The reduced opportunity to learn and experience common thematic associates of food perpetuates unfamiliarity in food-based situations and thus drives food rejection tendencies. As the first study to detect a negative relationship between thematic food categorization and food rejection, these results suggest that enriching thematic food category knowledge in young children could be an efficient strategy to foster dietary variety. Future research should address the specific associations children with food rejection tendencies rely upon when making inferences about food categories and, most importantly, how improving children's food knowledge of the varieties of thematic associations may promote dietary variety.

## Data Availability Statement

The datasets presented in this study can be found in online repositories. The names of the repository/repositories and accession number(s) can be found at: https://doi.org/10.5281/zenodo.3749248.

## Ethics Statement

Ethical review and approval was not required for the study on human participants in accordance with the local legislation and institutional requirements. Written informed consent to participate in this study was provided by the participants' legal guardian/next of kin. Written informed consent was obtained from the minor(s)' legal guardian/next of kin for the publication of any potentially identifiable images or data included in this article.

## Author Contributions

AP, J-PT, and JL contributed to the design, implementation of the research, and to the writing of the manuscript. AP collected the data and performed the analyses. All authors contributed to the article and approved the submitted version.

## Conflict of Interest

The authors declare that the research was conducted in the absence of any commercial or financial relationships that could be construed as a potential conflict of interest.

## Appendix

**Appendix A:** List of the complete stimuli sets.

	**Analogy Pair**		**Triad**		
	**A**	**B**	**C**	**D:Taxonomic**	**D:Thematic**
**Training Phase**					
Thematic Ex.1	Notebook	Pencil			
Thematic Ex.2	Bee	Honey			
Taxonomic Ex.1	Dog	Chimpanzee			
Taxonomic Ex.2	Necklace	Ring			
			Soccer Shoe	Rain Boots	Soccer Ball
**Test Phase**					
Thematic Ex.1	Ice Cream	Wafer Cone			
Thematic Ex.2	Pancakes	Chocolate Sauce			
Taxonomic Ex.1	Banana	Apple			
Taxonomic Ex.2	Sardine	Salmon			
			Lemon	Cherry	Fish
			Sausage	Steak	Mashed Potatoes
			Milk	Camembert	Cereal
			Spaghetti	Couscous	Bolognaise
			Chocolate	Sweets	Bread Roll
			Cheese Dessert	Hard Cheese	Sugar
			Grated Cheese	Milk	Macaroni
			Green Beans	Beetroot	Butter
			Grapefruit	Pear	Sugar
			Beef Patty	Chicken	Burger Bun
			Gherkin	Sweetcorn	Pâté
			Strawberry	Satsuma	Pastry
			Nuggets	Steak	Ketchup
			Cheese Slice	Yogurt	Sliced Bread
			Apple	Gooseberry	Puff Pastry
			Cheese Spread	Yogurt	Breadsticks
